# Knowledge, attitude, and practice towards postmenopausal osteoporosis among postmenopausal women: A cross-sectional study

**DOI:** 10.1016/j.pmedr.2025.103234

**Published:** 2025-09-08

**Authors:** Feng Xiong, Aoqi Zang, Zhe Xu, Qingyi Cao, Ziquan Shen, Feng Qian, Hui Xu

**Affiliations:** a Department of orthopedic, Bengbu First People's Hospital, Bengbu, AnHui 233000, China; b Department of Cardiology, Bengbu First People's Hospital, Bengbu, AnHui 233000, China; c Department of Anesthesiology, Bengbu First People's Hospital, Bengbu, AnHui 233000, China

**Keywords:** Postmenopausal osteoporosis, Cross-sectional study, Health education, Postmenopausal women, KAP

## Abstract

**Objective:**

Postmenopausal osteoporosis, affecting 33 % of women over 60 in China, increases fracture risks and significantly impacts quality of life. This condition leads to physical disability and psychological distress, underscoring the need for targeted interventions. This study aimed to assess the knowledge, attitude, and practice (KAP) of postmenopausal women concerning postmenopausal osteoporosis and explore associated factors.

**Methods:**

A cross-sectional study was conducted from June 29 to September 8, 2024, at the First People's Hospital of Bengbu City using a self-administered questionnaire to assess KAP, and data were analyzed using descriptive statistics and nonparametric tests.

**Results:**

A total of 459 valid participants in the study, of whom 166 (36.17 %) were diagnosed with postmenopausal osteoporosis. Among those diagnosed with osteoporosis, 65 (39.16 %) received systematic treatment. The mean scores for knowledge, attitude, and practice were 10.03 ± 5.47 (range: 0–28), 28.03 ± 3.07 (range: 8–40), and 21.87 ± 5.10 (range: 7–35), respectively.

**Conclusion:**

While postmenopausal women demonstrated insufficient knowledge and inadequate practices, targeted educational efforts may help improve osteoporosis-related behaviors and outcomes. These findings highlight the need for targeted educational interventions to improve osteoporosis-related knowledge and encourage proactive behaviors in managing the disease.

## Highlights

• One third of postmenopausal women in China are affected.
• More than one third diagnosed with postmenopausal osteoporosis.
• Knowledge impacts attitude and practice significantly.
• Postmenopausal women had poor knowledge but showed positive attitudes.
• Need for targeted educational interventions emphasized.

## 1 Introduction

Postmenopausal osteoporosis is a prevalent condition that significantly increases the risk of fractures and adversely impacts the quality of life in women. The prevalence of osteoporosis among postmenopausal women is a growing concern, with a large portion of this population at risk of developing the condition. Various factors, including age, lifestyle, and genetic predisposition, contribute to the incidence of osteoporosis in postmenopausal women(Ahn et al., 2012). Osteoporosis affects approximately 33 % of women over 60 years old in China, with postmenopausal osteoporosis being the most common type(Clynes et al., 2020; Rolvien et al., 2020). Fractures resulting from this condition not only lead to physical disabilities but also contribute to psychological distress, further reducing the quality of life(Gao and Zhao, 2023). Beyond physical health, osteoporosis also increases dependency and limits social engagement, both of which are vital to overall well-being(Nair et al., 2024; Ünver et al., 2024). Women with postmenopausal osteoporosis often experience a significantly lower quality of life compared to those with normal bone density. Chronic diseases, a history of fractures, and psychosocial factors are key contributors to this diminished quality of life(Bonaccorsi et al., 2021; Górczewska and Jakubowska-Pietkiewicz, 2022). Addressing the prevalence of osteoporosis in this demographic is crucial for improving health outcomes and enhancing the overall well-being of postmenopausal women in China.

The knowledge attitude practice (KAP) model is essential in shaping health behaviors and is frequently applied in healthcare to assess and improve knowledge, attitudes, and practices within a population. The KAP questionnaire, grounded in this model, is often used to evaluate the population's understanding and acceptance of health-related content, thereby providing insights into public health needs(Li et al., 2020). The model operates on the principle that increased knowledge positively influences attitudes, which in turn affect individual practices(Khalid et al., 2022). Identifying factors associated with KAP is essential for designing more targeted and effective interventions. Given the high prevalence of postmenopausal osteoporosis in China, targeted interventions and awareness programs are necessary to reduce fracture risks and improve the quality of life for affected women(Bartosch et al., 2021). This study aims to assess the knowledge, attitudes, and practices of postmenopausal women regarding osteoporosis and identify associated factors.

## 2 Methods

### 2.1 Study design and participants

This cross-sectional study was conducted from June 29 to September 8, 2024, at the First People's Hospital of Bengbu City. The study included postmenopausal women who were capable of independently understanding and answering the questionnaire. Women who were not postmenopausal, as well as individuals with mental health issues or emotional instability that impaired their ability to comprehend the survey, were excluded. Ethical approval was obtained from the hospital's ethics committee under approval number BBYY2024067, and informed consent was obtained from all participants.

### 2.2 Questionnaire design

A self-administered questionnaire was reviewed by five experts in the field. A small-scale pilot study (47 questionnaires) was conducted, yielding a Cronbach's α coefficient of 0.91, indicating good internal consistency.

The questionnaire was distributed through four methods: 1) sharing osteoporosis-related articles containing the questionnaire link on the hospital's WeChat public platform; 2) distributing the questionnaire's Quick Response code in WeChat groups of retired medical and nursing staff; 3) offering on-site Quick Response code scanning for participants to complete the questionnaire during the hospital's outreach clinic activities; and 4) having the head nurses assist eligible inpatients in completing the questionnaire by scanning the Quick Response code one-on-one.

The questionnaire content covered four aspects: demographic characteristics, knowledge dimension, attitude dimension, and practice dimension. The knowledge dimension included 15 items, with one trap question (the fifteenth) excluded from analysis. Other items were scored as two points for ‘very familiar’, one point for ‘have heard of it’, and zero points for ‘unclear’, with a score range of 0–28. The attitude dimension contained eight items using a five-point Likert scale, with values ranging from 5 (strongly agree) to 1 (strongly disagree), resulting in a score range of 8–40. Among them, items A5 (“I believe regular exercise does not significantly help maintain bone health”), A6 (“I believe smoking and alcohol consumption are unrelated to osteoporosis”), and A8 (“I would be concerned about the side effects of long-term medication use”) were reverse-coded, with the scoring assigned from 1 to 5 for responses A to E. This reverse coding was correctly applied during the data analysis. The practice dimension included seven items using a five-point Likert scale, with values ranging from 5 (always) to 1 (never), resulting in a score range of 7–35.

### 2.3 Statistical analysis

Continuous variables were presented as means and standard deviations (SD), while categorical variables were expressed as frequencies and percentages (n, %). Since the data did not meet the assumption of normality, the Mann-Whitney *U* test and Kruskal-Wallis H test were used to compare KAP scores across different demographic groups. A score exceeding 70 % of the total in KAP domains was defined as indicating adequate knowledge, a positive attitude, and proactive practice. A two-sided *P*-value of less than 0.05 was considered statistically significant. For pairwise comparisons following Kruskal-Wallis tests, Bonferroni correction was applied to adjust for multiple testing. Data analysis was performed using SPSS 27.0 (IBM, Armonk, NY, USA) and AMOS 26.0 (IBM, Armonk, NY, USA).

## 3 Results

### 3.1 Demographic characteristics

**Figure S1:**
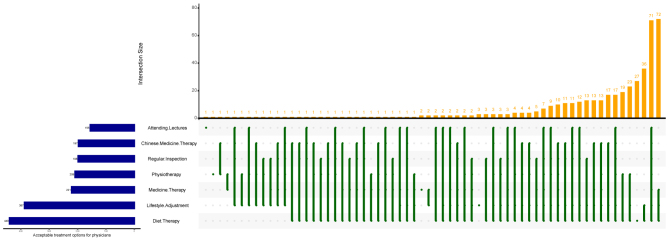
Treatment types received by postmenopausal women diagnosed with osteoporosis in Bengbu, China (July–September 2024). Footnote: Intersection size refers to the number of participants corresponding to each set or dot matrix combination shown below the bars.

A total of 575 questionnaires were enrolled and 459 questionnaires remained after cleaning, with a validity rate of 79.83 %. Among the 459 postmenopausal women included in this study, 444 (96.73 %) were married. The participants had a mean age of 59.74 ± 7.96 years, and the mean age of menopause was 50.18 ± 2.70 years. In terms of education, 363 (79.08 %) had completed middle school or below, and 166 (36.17 %) were diagnosed with postmenopausal osteoporosis. Of those with osteoporosis, 65 (39.16 %) received systematic treatment, and 101 (60.84 %) had used medication for the condition. The mean scores for knowledge, attitude, and practice were 10.03 ± 5.47, 28.03 ± 3.07, and 21.87 ± 5.10, respectively. Demographic analyses showed significant differences in knowledge, attitude, and practice scores based on education level (*P* < 0.01 for all), household monthly income (P < 0.01 for all), number of children (P < 0.01 for all), and current diagnosis of postmenopausal osteoporosis (*P* < 0.001 for all). In addition, type of menopause (*P* = 0.01) and family history of postmenopausal osteoporosis (P < 0.01) also showed differences in attitudes and knowledge, respectively (Table 1). Post-hoc comparisons with Bonferroni correction revealed significant differences in KAP scores across education level, household income, and number of children. Adjusted *p*-values are shown in Table 2**.** Fig. S1 illustrated the acceptance of various treatment among postmenopausal women with osteoporosis. Diet therapy and lifestyle adjustment were the most accepted, with 440 and 387 participants respectively. In contrast, attending lectures was the least accepted, with only 155 participants. The top intersection of the figure showed that 71 participants were receptive to all treatment modalities, while 34 were receptive to only one modality.

### 3.2 The distribution of KAP

**Fig. 1:**
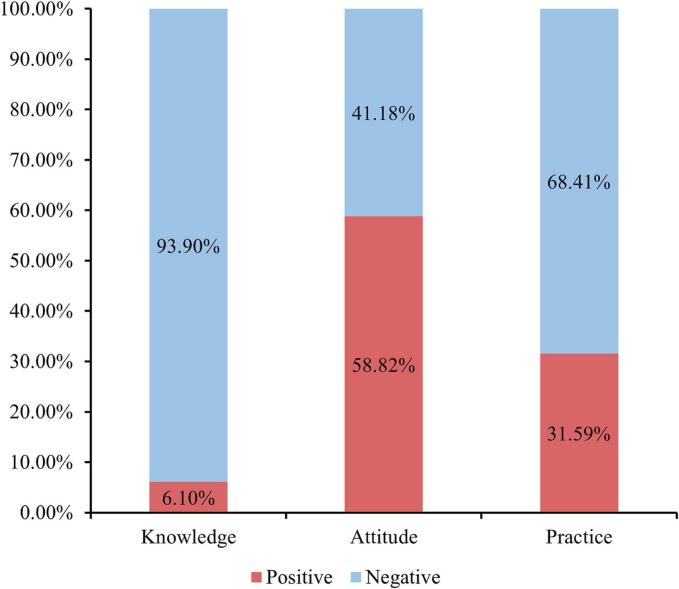
Number of women with adequate knowledge, positive attitude, and proactive behavior in Bengbu, China (July–September 2024).

The distribution of knowledge dimensions shown that the three questions with the highest number of participants choosing the “Not sure” option were “Bisphosphonates can inhibit osteoclast activity, calcitonin can prevent osteoporosis and relieve pain, and estrogen receptor antagonists and hormone replacement therapy are also used for treatment.” (K7) with 57.3 %, “The development of postmenopausal osteoporosis is related to unhealthy habits such as smoking, alcohol consumption, and caffeine intake.” (K12) with 48.8 %, and “Although postmenopausal osteoporosis is not a typical genetic disease, genetic factors do influence bone density and susceptibility to osteoporosis to some extent.” (K11) with 48.58 %. Responses to the attitude dimension showed that 37.25 % believed that regular exercise did not significantly help in maintaining bone health (A5), and 22.22 % believed that smoking and alcohol consumption were not associated with osteoporosis. In addition, 52.51 % were concerned about the side effects of long-term use of medication for osteoporosis (A8). Responses to the practice dimension showed that 21.79 % never avoid excessive alcohol consumption and refrain from smoking to reduce the risk of osteoporosis (P4), 17.21 % never had regular bone density tests (P5), and 15.03 % never reported any osteoporosis-related symptoms to their doctors (P6) (Table 3). According to the 70 % threshold, participants scoring ≥19.6 out of 28 in knowledge, ≥28 out of 40 in attitude, and ≥ 24.5 out of 35 in practice were classified as having adequate levels in each respective domain. A total of 28 participants (6.10 %) had adequate knowledge, 270 (58.82 %) had a positive attitude, and 145 (31.59 %) demonstrated proactive practice (Fig. 1).

**Table 1:** Demographic characteristics and questionnaire scores among postmenopausal women in Bengbu, China (July–September 2024).⁎

Variables	N (%)	Knowledge, mean ± SD	*P*	Attitude, mean ± SD	*P*	Practice, mean ± SD	*P*
*N* = 459		10.03 ± 5.47		28.03 ± 3.07		21.87 ± 5.10	
Age	59.74 ± 7.96						
Age at menopause	50.18 ± 2.70						
Marital status			0.83		0.11		0.70
Married	444 (96.73)	9.96 ± 5.31		27.99 ± 3.04		21.86 ± 5.09	
Single/divorced/widowed	15 (3.27)	12.20 ± 9.02		29.47 ± 3.68		22.13 ± 5.49	
Education			<0.01		<0.01		<0.01
Middle school and below	363 (79.08)	9.20 ± 4.81		27.44 ± 2.70		21.32 ± 5.04	
High school/vocational school	43 (9.37)	12.58 ± 6.75		29.40 ± 3.26		23.67 ± 4.32	
Associate degree	33 (7.19)	12.85 ± 5.72		30.67 ± 2.99		23.55 ± 5.17	
Bachelor's degree and above	20 (4.36)	15.10 ± 7.40		31.50 ± 3.82		25.20 ± 5.17	
Household monthly income			<0.01		<0.01		<0.01
<5000	319 (69.50)	9.05 ± 4.83		27.24 ± 2.76		20.98 ± 5.04	
5000–10,000	103 (22.44)	11.85 ± 6.26		29.35 ± 2.77		23.37 ± 4.36	
>10,000	37 (8.06)	13.43 ± 5.77		31.19 ± 3.25		25.35 ± 5.19	
Type of menopause			0.23		0.01		0.57
Natural menopause	444 (96.73)	9.95 ± 5.40		27.96 ± 3.03		21.84 ± 5.13	
Induced menopause (surgery, chemotherapy, etc)	15 (3.27)	12.47 ± 7.05		30.13 ± 3.64		22.80 ± 4.09	
Current parental status			0.63		0.89		0.54
Yes	418 (91.07)	9.97 ± 5.37		28.04 ± 3.03		21.83 ± 5.16	
No	41 (8.93)	10.68 ± 6.43		28.02 ± 3.55		22.29 ± 4.45	
Number of children			<0.01		<0.01		<0.01
One	95 (22.73)	12.38 ± 6.17		29.53 ± 3.28		23.91 ± 4.56	
Two	202 (48.33)	9.12 ± 4.58		27.60 ± 2.81		20.91 ± 5.17	
Three or more	121 (28.95)	9.50 ± 5.41		27.60 ± 2.82		21.74 ± 5.16	
Use of estrogen			0.05		0.39		0.80
Yes	72 (15.69)	11.88 ± 7.02		28.38 ± 3.33		22.03 ± 5.20	
No	387 (84.31)	9.69 ± 5.07		27.97 ± 3.02		21.84 ± 5.09	
Family history of postmenopausal osteoporosis			<0.01		0.07		0.19
Yes	91 (19.83)	11.90 ± 6.33		28.58 ± 3.16		22.77 ± 5.25	
No	228 (49.67)	9.96 ± 5.05		27.67 ± 2.85		21.60 ± 4.98	
Unclear	140 (30.50)	8.94 ± 5.24		28.28 ± 3.30		21.72 ± 5.16	
Current diagnosis of postmenopausal osteoporosis:			0.01		<0.01		<0.01
Yes	166 (36.17)	11.14 ± 5.76		28.79 ± 3.00		23.01 ± 4.80	
No	293 (63.83)	9.40 ± 5.20		27.61 ± 3.04		21.23 ± 5.16	
Systematic treatment for postmenopausal osteoporosis			0.48		0.07		0.76
Yes	65 (39.16)	11.74 ± 6.40		28.37 ± 3.18		23.37 ± 5.52	
No	101 (60.84)	10.76 ± 5.30		29.06 ± 2.85		22.77 ± 4.29	
Use of osteoporosis medication			0.39		0.67		0.31
Yes	101 (60.84)	11.34 ± 5.58		28.71 ± 2.90		23.37 ± 4.78	
No	65 (39.16)	10.85 ± 6.05		28.91 ± 3.16		22.45 ± 4.82	

Currency: Chinese Yuan (CNY); approximate equivalent: 1 CNY ≈ 0.14 USD.

*P*-values were calculated using the Mann–Whitney *U* test or Kruskal–Wallis H test, depending on the number of comparison groups.

⁎ Systematic treatment refers to comprehensive management strategies including medications, calcium/vitamin D supplementation, and lifestyle modifications such as diet and exercise.

**Table 2:** Post-hoc comparison of questionnaire scores by sociodemographic groups among postmenopausal women in Bengbu, China (July–September 2024).

		Knowledge		Attitude		Practice	
		P	Bonferroni P	P	Bonferroni P	P	Bonferroni P
Education	Middle school and below VS High school/vocational school	0.01	0.01	<0.001	0.01	0.01	0.03
	Middle school and below VS Associate degree	<0.01	<0.01	<0.01	<0.01	0.03	0.17
	Middle school and below VS Bachelor's degree and above	<0.01	0.01	<0.01	<0.01	0.01	0.01
	High school/vocational school VS Associate degree	0.38	1.00	0.11	0.67	0.82	1.00
	High school/vocational school VS Bachelor's degree and above	0.19	1.00	0.11	0.63	0.31	1.00
	Associate degree VS Bachelor's degree and above	0.59	1.00	0.80	1.000	0.25	1.00
Household monthly income	<5000 VS 5000–10,000	<0.01	<0.01	<0.01	<0.01	<0.01	<0.01
	<5000 VS >10,000	<0.01	<0.01	<0.01	<0.01	<0.01	<0.01
	5000–10,000 VS >10,000	0.05	0.13	0.02	0.05	0.08	0.22
Number of children	One VS Two	<0.01	<0.01	<0.01	<0.01	<0.01	<0.01
	One VS Three or more	<0.01	<0.01	<0.01	<0.01	0.01	0.02
	Two VS Three or more	0.75	1.00	0.76	1.00	0.08	0.24
Family history of postmenopausal osteoporosis	Yes VS No	0.02	0.05	/	/	/	/
	Yes VS Unclear	<0.01	<0.01	/	/	/	/
	No VS Unclear	0.03	0.09	/	/	/	/

P-values were derived from pairwise comparisons following Kruskal–Wallis tests. Bonferroni-corrected p-values were calculated based on the number of comparisons conducted for each sociodemographic variable: education (6 comparisons), household income (3 comparisons), number of children (3 comparisons), and family history (3 comparisons).

**Table 3:** Response distribution of knowledge, attitude, and practice items among postmenopausal women in Bengbu, China (July–September 2024).

Knowledge		N (%)			
		Very familiar		Heard of it	Unclear
1. Postmenopausal osteoporosis is mainly caused by a decrease in estrogen levels. The reduction in estrogen weakens the inhibition of osteoclast activity, accelerating bone loss.		33 (7.19)		236 (51.42)	190 (41.39)
2. The period 5 to 15 years after menopause (approximately ages 51 to 70) is a high-risk period for postmenopausal osteoporosis.		30 (6.54)		226 (49.24)	203 (44.23)
3. Common symptoms of postmenopausal osteoporosis include lower back pain, height loss, hunchback, and an increased risk of fractures (such as vertebral compression fractures and distal radius fractures).		37 (8.06)		279 (60.78)	143 (31.15)
4. Lower back pain is usually diffuse, persistent, and dull, and may be aggravated by changes in posture, carrying loads, or prolonged activity, with slight relief during rest.		32 (6.97)		273 (59.48)	154 (33.55)
5. Preventive measures for postmenopausal osteoporosis include a balanced diet (increasing calcium and vitamin D intake), regular exercise (such as walking, jogging, Tai Chi), adequate sunlight exposure, and regular bone density checks.		63 (13.73)		306 (66.67)	90 (19.61)
6. Calcium and vitamin D3 supplements are a basic treatment that helps slow down bone loss.		69 (15.03)		278 (60.57)	112 (24.4)
7. Bisphosphonates can inhibit osteoclast activity, calcitonin can prevent osteoporosis and relieve pain, and estrogen receptor antagonists and hormone replacement therapy are also used for treatment.		24 (5.23)		172 (37.47)	263 (57.30)
8. Exercise stimulates bone formation through muscle activity-induced stress, increasing bone mineral content. Older adults are encouraged to engage in physical activity or exercise within their capacity.		52 (11.33)		256 (55.77)	151 (32.90)
9. Outdoor sunlight exposure not only aids in the synthesis of vitamin D but is also recommended for at least 30 min per session to promote calcium absorption and bone health.		90 (19.61)		275 (59.91)	94 (20.48)
10. Using estrogen can somewhat delay or treat postmenopausal osteoporosis.		28 (6.10)		227 (49.46)	204 (44.44)
11. Although postmenopausal osteoporosis is not a typical genetic disease, genetic factors do influence bone density and susceptibility to osteoporosis to some extent.		22 (4.79)		214 (46.62)	223 (48.58)
12. The development of postmenopausal osteoporosis is related to unhealthy habits such as smoking, alcohol consumption, and caffeine intake.		33 (7.19)		202 (44.01)	224 (48.8)
13. During pregnancy and breastfeeding, the mother's body undergoes a series of physiological changes, including the redistribution of calcium and other minerals to support fetal development and milk production. During this period, insufficient calcium intake or imbalanced calcium metabolism may affect a woman's bone density.		35 (7.63)		247 (53.81)	177 (38.56)
14. Treatment of postmenopausal osteoporosis should adopt a comprehensive approach, combining medication, lifestyle adjustments, and regular monitoring to achieve optimal outcomes.		33 (7.19)		252 (54.9)	174 (37.91)
Attitude	N (%)				
	Strongly agree	Agree	Neutral	Disagree	Strongly disagree
1. I believe postmenopausal osteoporosis is a common health issue among postmenopausal women.	33 (7.19)	240 (52.29)	165 (35.95)	19 (4.14)	2 (0.44)
2. I believe early diagnosis and treatment can effectively control postmenopausal osteoporosis.	47 (10.24)	252 (54.9)	153 (33.33)	7 (1.53)	/
3. I think regular bone density check-ups are crucial for the prevention of osteoporosis.	50 (10.89)	233 (50.76)	170 (37.04)	5 (1.09)	1 (0.22)
4. I am willing to increase my intake of calcium and vitamin D to prevent osteoporosis.	59 (12.85)	264 (57.52)	131 (28.54)	5 (1.09)	/
5. I believe regular exercise do not significantly help maintain bone health.	28 (6.1)	171 (37.25)	165 (35.95)	85 (18.52)	10 (2.18)
6. I believe smoking and alcohol consumption are unrelated to osteoporosis.	15 (3.27)	102 (22.22)	179 (39)	146 (31.81)	17 (3.70)
7. If diagnosed with osteoporosis, I would actively seek medical intervention.	56 (12.2)	248 (54.03)	144 (31.37)	10 (2.18)	1 (0.22)
8. If diagnosed with osteoporosis, I would be concerned about the side effects of long-term medication use.	20 (4.36)	241 (52.51)	171 (37.25)	26 (5.66)	1 (0.22)
Practice	N (%)				
	Always	Often	Sometimes	Rarely	Never
1. I ensure that my daily diet includes enough calcium and vitamin D.	32 (6.97)	198 (43.14)	188 (40.96)	31 (6.75)	10 (2.18)
2. I engage in outdoor activities regularly to increase exposure to natural sunlight and promote vitamin D production.	49 (10.68)	214 (46.62)	145 (31.59)	22 (4.79)	29 (6.32)
3. I participate in exercises that help increase bone density, such as walking, jogging, or weight training.	37 (8.06)	171 (37.25)	169 (36.82)	41 (8.93)	41 (8.93)
4. I avoid excessive alcohol consumption and refrain from smoking to reduce the risk of osteoporosis.	99 (21.57)	102 (22.22)	99 (21.57)	59 (12.85)	100 (21.79)
5. I regularly undergo bone density testing, at least once every two years.	22 (4.79)	75 (16.34)	169 (36.82)	114 (24.84)	79 (17.21)
6. I report any osteoporosis-related symptoms, such as fractures, height loss, or back pain, to my doctor.	17 (3.7)	87 (18.95)	173 (37.69)	113 (24.62)	69 (15.03)
7. I take precautions to ensure home safety and prevent falls, such as installing handrails, removing obstacles in the home, and using proper posture when doing household chores or lifting heavy objects to protect my bones.	34 (7.41)	151 (32.9)	174 (37.91)	57 (12.42)	43 (9.37)

## 4 Discussion

Postmenopausal women demonstrated insufficient knowledge, positive attitudes, and suboptimal practices regarding postmenopausal osteoporosis. To improve osteoporosis management, targeted educational interventions should be implemented to enhance both knowledge and practical preventive behaviors in postmenopausal women.

This lack of comprehensive knowledge, combined with inadequate practices, might be a significant factor contributing to the ongoing challenges in managing osteoporosis effectively among postmenopausal women. It is evident from the current study that while participants hold positive attitudes towards managing their condition, such as agreeing on the importance of treatment and exercise, they often fail to translate these attitudes into action, as reflected by low practice scores. This pattern is consistent with other research, which suggests that positive health attitudes alone are insufficient to drive change unless coupled with better education and accessible interventions(Aldhamy et al., 2023; Tahani and Manesh, 2021).

The low levels of knowledge among postmenopausal women about key aspects of osteoporosis prevention and treatment are concerning. For instance, a substantial portion of participants were unsure about the role of bisphosphonates and the influence of lifestyle factors like smoking and alcohol consumption on bone health. These misconceptions align with the findings of previous studies where women reported uncertainty or a lack of awareness about common osteoporosis treatments and prevention strategies(Arceo-Mendoza and Camacho, 2021; Brown, 2021). Such gaps in knowledge likely contribute to the poor adherence to recommended practices, such as bone density testing and symptom reporting, both of which are essential for the timely diagnosis and management of osteoporosis. A similar lack of knowledge was also identified in other studies, where participants expressed confusion regarding the effects of hormone therapy and the link between osteoporosis and genetic predisposition, further emphasizing the need for better educational interventions targeting postmenopausal populations(Ayers et al., 2023; Humphrey et al., 2023).

The significant differences in knowledge, attitude, and practice scores across various demographic variables shed light on the complex interplay between socio-demographic factors and osteoporosis management. Education level was one of the most significant predictors of KAP scores in this study, with women who had higher educational qualifications consistently scoring better across all three dimensions. This observation is consistent with findings from other studies, which have repeatedly demonstrated that higher education is associated with better health literacy, higher engagement in preventive health behaviors, and improved outcomes in managing chronic diseases(Chen et al., 2021; Nutbeam and Lloyd, 2021). For instance, a higher level of education often translates into greater access to health information, a better understanding of medical advice, and a stronger capacity to engage in preventive behaviors.

Conversely, women with lower education levels tended to score lower in all KAP dimensions, which may be indicative of barriers to accessing health information or understanding medical guidance. This finding underscores the importance of tailoring educational interventions to account for varying levels of literacy and health comprehension. Implementing health education campaigns that use simple language, visual aids, and culturally sensitive materials may be essential in bridging the knowledge gap for this population(Pan, 2023; Stock et al., 2022).

Household income also played a significant role, with higher-income participants reporting better KAP scores. This is in line with previous studies, where financial resources were linked to better access to healthcare services, medications, and preventive care options, such as regular bone density testing(Berezowski et al., 2023; Rice et al., 2020). The financial burden associated with managing osteoporosis, particularly in resource-limited settings, might explain why lower-income women are less likely to engage in preventive practices. Providing affordable screening programs, subsidized medications, and community-based interventions can help mitigate these disparities, ensuring that women from lower-income brackets can still access essential care(Maganty et al., 2023; Staats et al., 2022).

Interestingly, induced menopause was associated with higher attitude scores, likely due to the increased medical attention and follow-up care these women received after surgery or chemotherapy. Women undergoing induced menopause are typically more exposed to healthcare providers, which might positively influence their health attitudes(Echeverria et al., 2021; Maki and Jaff, 2022). However, this heightened awareness did not translate into significantly better practice scores, suggesting that while attitudes towards treatment may be favorable, structural barriers—such as access to care or motivation—might still prevent these women from fully implementing recommended health behaviors. Future studies should explore the reasons behind this discrepancy to better tailor interventions for women with induced menopause, ensuring that positive attitudes lead to practical changes in behavior.

The distribution of knowledge, attitude, and practice scores reflects several concerning patterns. In the knowledge dimension, many participants were unsure about fundamental aspects of osteoporosis management, such as the role of bisphosphonates, the genetic factors associated osteoporosis, and the link between lifestyle choices like smoking and bone health. Such knowledge gaps are critical, as they directly impact practice, such as adherence to medication, regular bone density screening, and reporting osteoporosis-related symptoms. For example, a considerable number of participants did not undergo regular bone density tests, a practice that is essential for monitoring bone health and preventing fractures. This is consistent with findings from similar studies where low adherence to regular screening was attributed to a lack of knowledge about its importance(Chaemsaithong et al., 2022; Cross et al., 2020).

In terms of attitudes, the study found that while most participants held positive views about the importance of early diagnosis and treatment, many were less convinced about the role of regular exercise and lifestyle modifications in preventing osteoporosis. Similarly, a notable proportion of participants expressed concerns about the long-term use of medication for osteoporosis, which might explain the reluctance to engage in systematic treatment or medication adherence. Addressing these misconceptions through clear communication about the benefits and risks of osteoporosis medication is critical. Healthcare providers should be encouraged to offer detailed counseling sessions to clarify these concerns and provide reassurance to patients(Chow et al., 2017; Liu et al., 2023).

Given the suboptimal practice scores, especially in areas such as avoiding smoking, alcohol consumption, and reporting symptoms, targeted interventions are necessary. First, expanding community outreach programs that focus on lifestyle modifications—such as smoking cessation workshops, physical activity programs tailored to older women, and educational sessions on diet and bone health—may significantly enhance engagement in preventive practices. Offering accessible bone density testing services, particularly in rural or lower-income areas, may also increase adherence to regular screening recommendations. Further, integrating osteoporosis education into routine healthcare visits, where women can receive direct guidance from their healthcare providers, could help reinforce the importance of proactive bone health management(Calaf-Alsina et al., 2023; Morin et al., 2023).

This study has several limitations. First, the use of online questionnaires may have introduced selection bias, as participants with internet access and higher health literacy may have been more likely to participate. Second, as with all self-administered survey studies, the questionnaire method is subject to inherent limitations such as response bias, social desirability bias, and potential misunderstanding of medical terminology by participants, even though a pilot study was conducted. Third, the cross-sectional design limits the ability to draw causal inferences about the observed relationships between knowledge, attitude, and practice. Finally, this study was conducted at a single hospital, which may affect the generalizability of the findings to other settings or populations.

## 5 Conclusions

In conclusion, postmenopausal women demonstrated insufficient knowledge, positive attitudes, and suboptimal practices regarding postmenopausal osteoporosis. Enhancing educational efforts to improve knowledge may lead to more proactive behaviors in the prevention and management of osteoporosis, ultimately improving patient outcomes.

The following are the supplementary data related to this article.

Supplementary material Supplementary material

## CRediT authorship contribution statement

**Feng Xiong:** Writing – review & editing, Writing – original draft, Investigation, Data curation. **Aoqi Zang:** Writing – review & editing, Writing – original draft, Investigation, Data curation. **Zhe Xu:** Writing – review & editing, Writing – original draft, Resources, Investigation. **Qingyi Cao:** Writing – review & editing, Writing – original draft, Software, Methodology. **Ziquan Shen:** Writing – review & editing, Writing – original draft, Software, Resources. **Feng Qian:** Writing – review & editing, Writing – original draft, Resources, Methodology. **Hui Xu:** Writing – review & editing, Writing – original draft, Project administration, Data curation, Conceptualization.

## Consent for publication

Not applicable.

## Ethics approval

The study was approved by Ethics Committee of Bengbu First People's Hospital (BBYY2024067). All participants were informed about the study protocol and provided written informed consent to participate in the study. I confirm that all methods were performed in accordance with the relevant guidelines. All procedures were performed in accordance with the ethical standards laid down in the 1964 Declaration of Helsinki and its later amendments.

## Funding

The study was supported by the Scientific Research Project of Bengbu Health Commission (BBWK2023A202) and the Natural Science Project of Bengbu Medical University(2023byzd196). The funders had no role in study design, data collection, and analysis, decision to publish, or preparation of the manuscript.

## Declaration of competing interest

The authors declare that they have no known competing financial interests or personal relationships that could have appeared to influence the work reported in this paper.

## Data Availability

All data generated or analyzed during this study are included in this published article.
